# Supplementary figures and images for: Multi-omics analyses of MEN1 missense mutations identify disruption of menin–MLL and menin–JunD interactions as critical requirements for molecular pathogenicity
Source: Epigenetics Chromatin. 2022 Aug 9;15:29. doi: 10.1186/s13072-022-00461-8 (PMC9361535; doi:10.1186/s13072-022-00461-8)

A

*MEN1/ ACTB RT-qPCR*

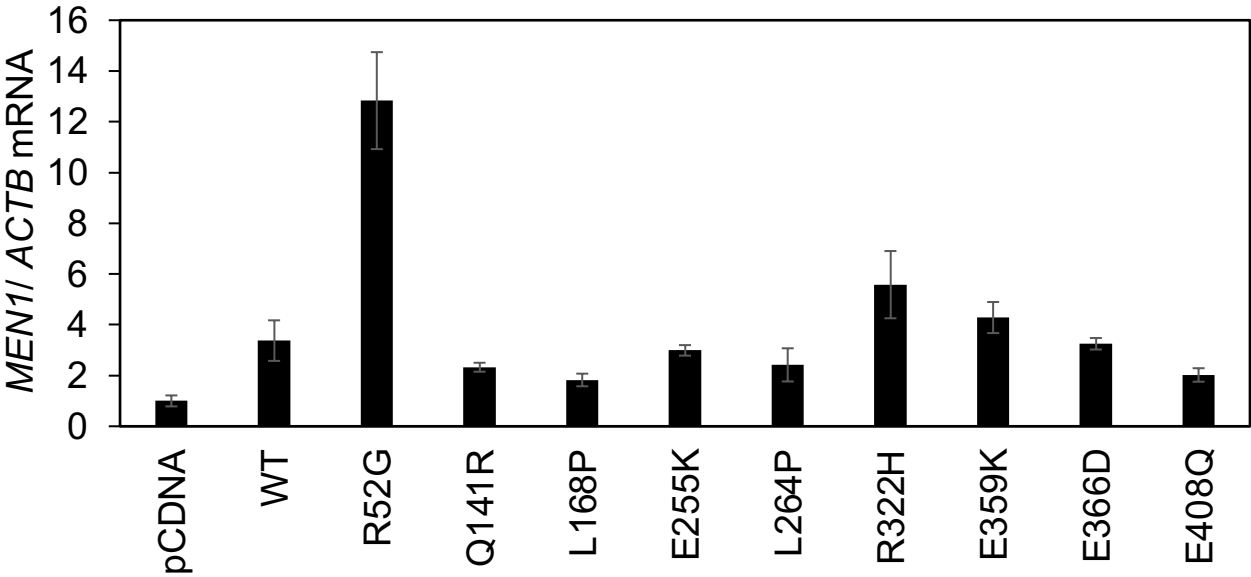

B

Menin protein/ *MEN1* mRNA

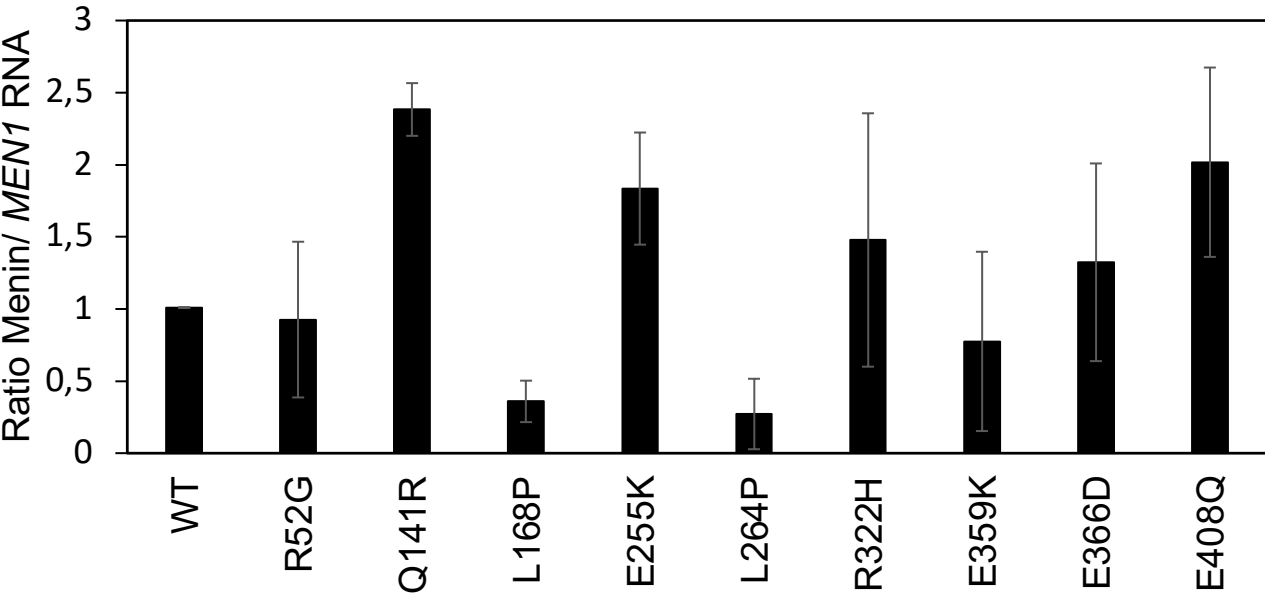

Supplement: Supplementary file 1 — Additional file 1: Figure S1. Quantification of menin mRNA and protein levels after transient transfections. Panel A. MEN1 mRNA expression was quantified by RT-qPCR and corrected for ACTB levels in pCDNA3.1MEN1 WT and mutant transfected into HEK293 cells. The endogenous MEN1/ACTB mRNA ratios in the control pCDNA transfected cells were set at 1. The experiments were performed in triplicate. Error bars indicate SE. Panel B. Ratios of menin expression quantitation and MEN1 mRNA levels determined by RT-qPCR in pCDNA3.1MEN1 wildtype and mutant transfected HEK293 cells, performed in triplicate. Error bars indicate SE. [file 13072_2022_461_MOESM1_ESM.pdf]

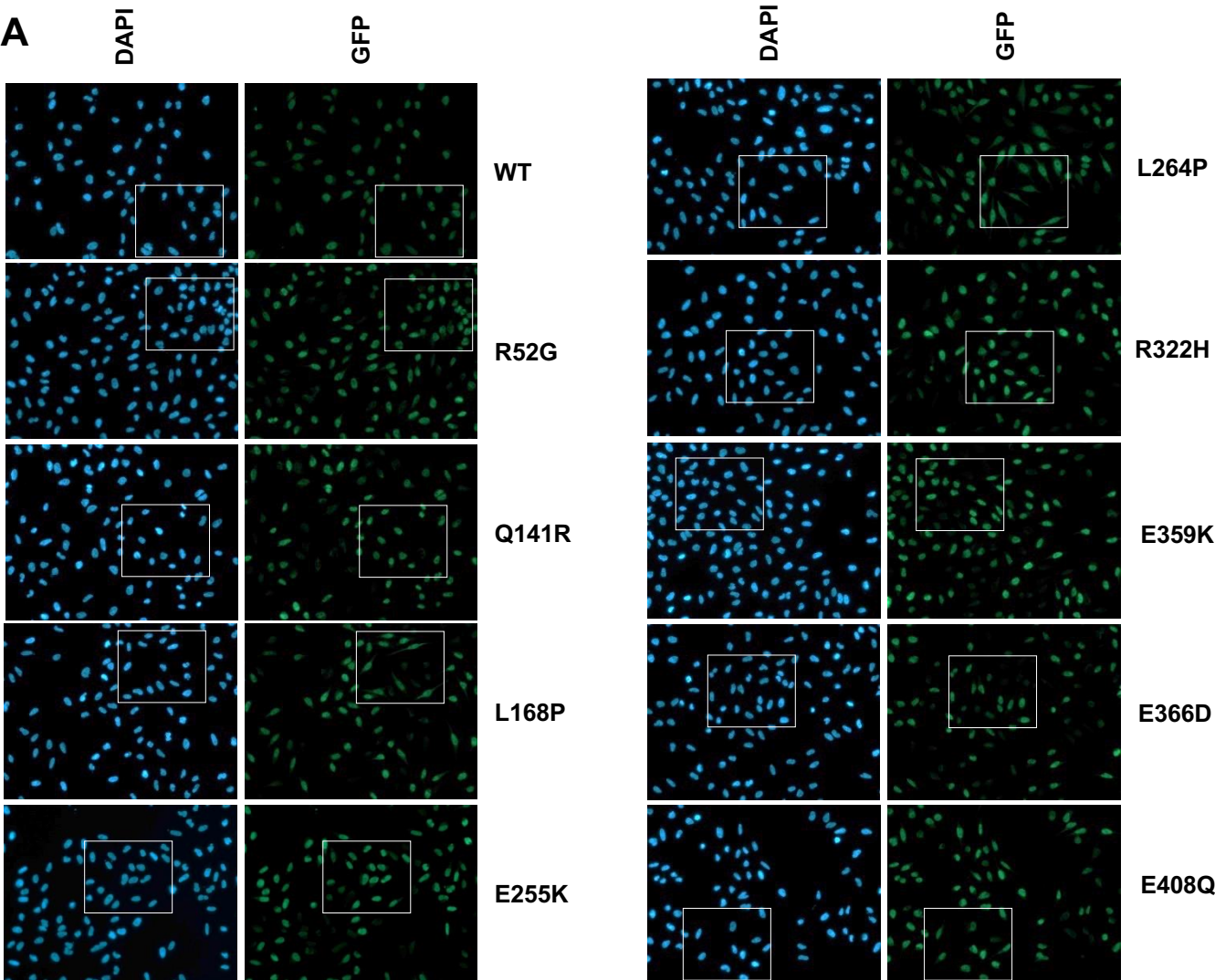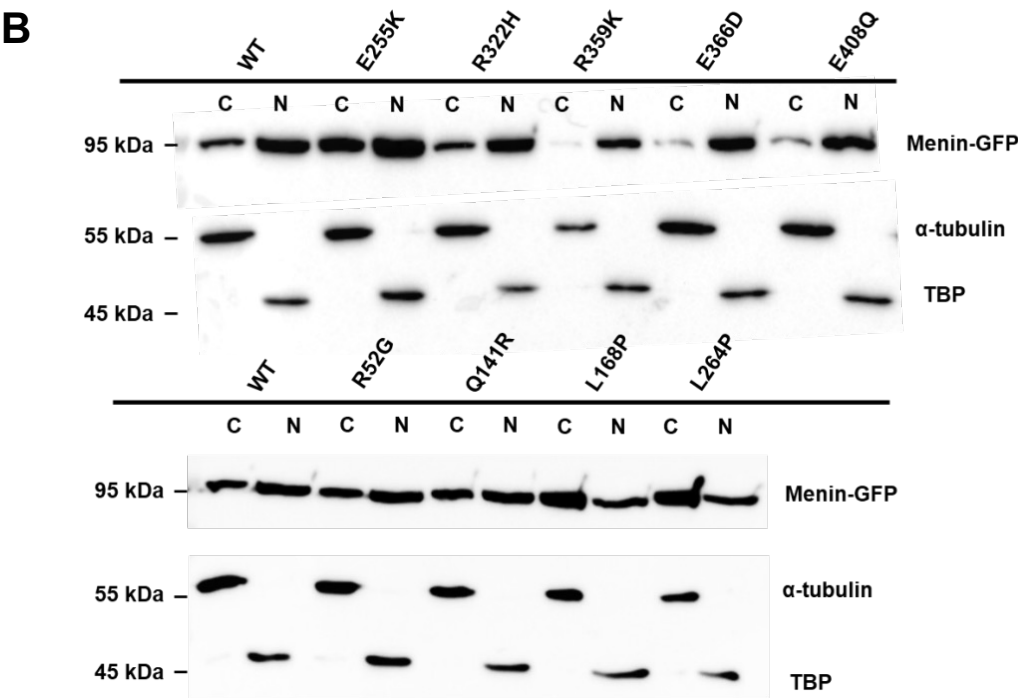

SUPPLEMENTARY FIGURE 2

Supplement: Supplementary file 2 — Additional file 2: Figure S2. Analysis of menin (mutant) proteins stably expressed in HeLa cells. Panel A. Fluorescence microscopy images of GFP-menin WT and mutant proteins stably expressed in HeLa cells stained with anti-GFP antibody. Zoomed regions were marked with white rectangles and are also provided in Fig. 2C. Panel B. Immunoblots of stable HeLa cell lines upon doxycycline for preparation of cytoplasmic and nuclear protein extracts. Expression of GFP-tagged menin was detected using GFP antibodies. α-tubulin and TBP antibodies were used as cytoplasmic and nuclear marker proteins and as loading controls. The position of co-migrated size markers are indicated on the left. [file 13072_2022_461_MOESM2_ESM.pdf]

**A**

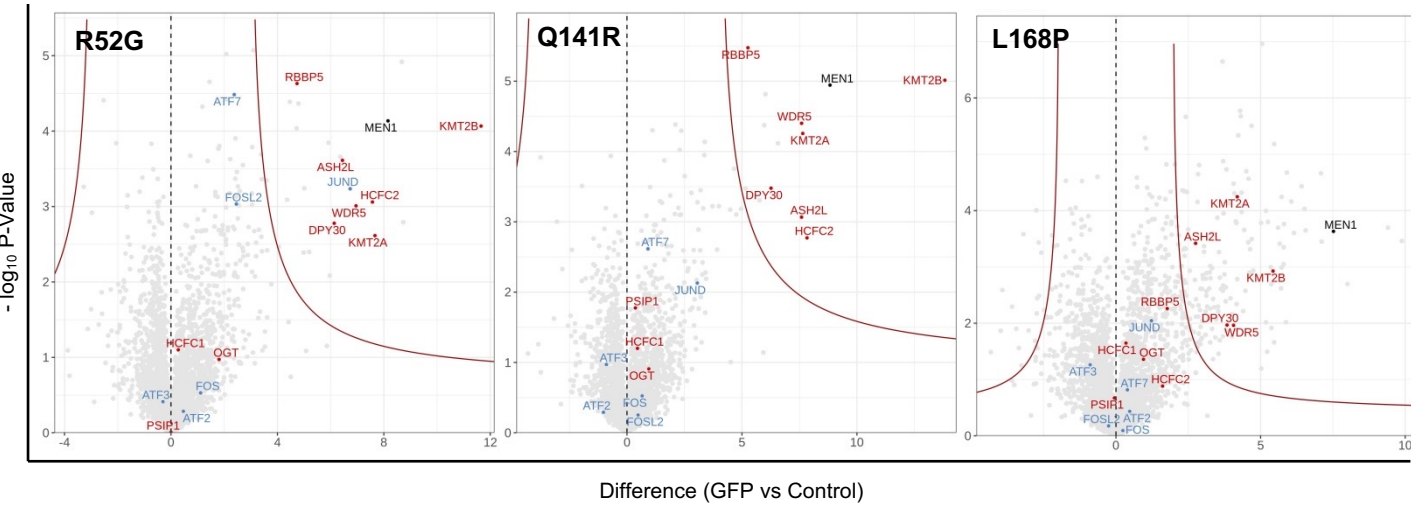

**B**

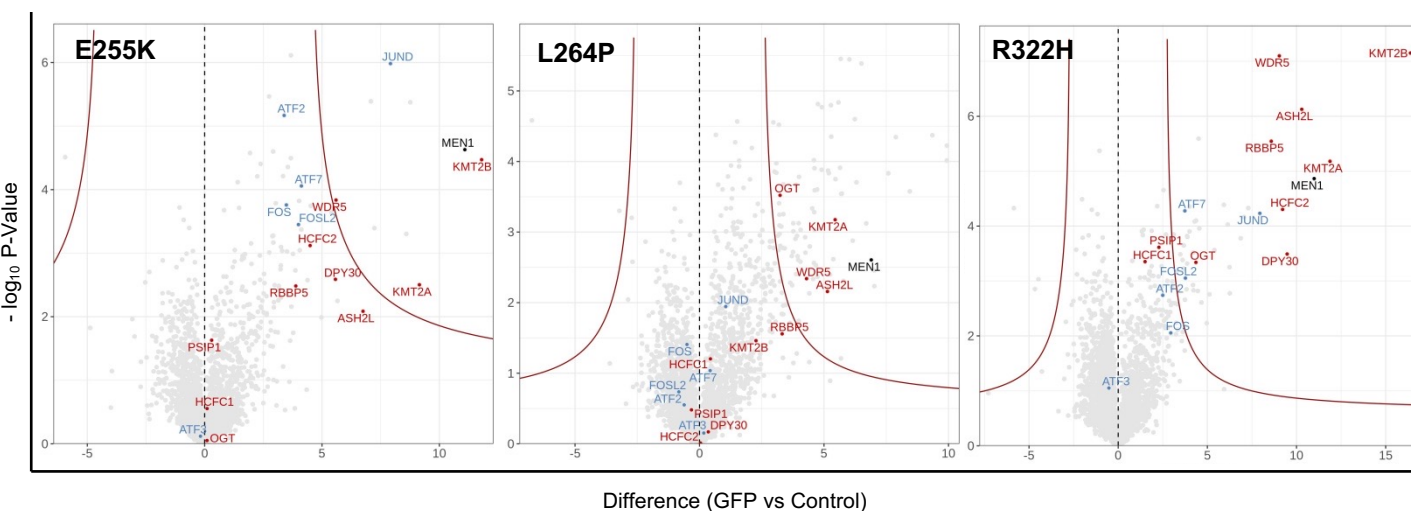

**C**

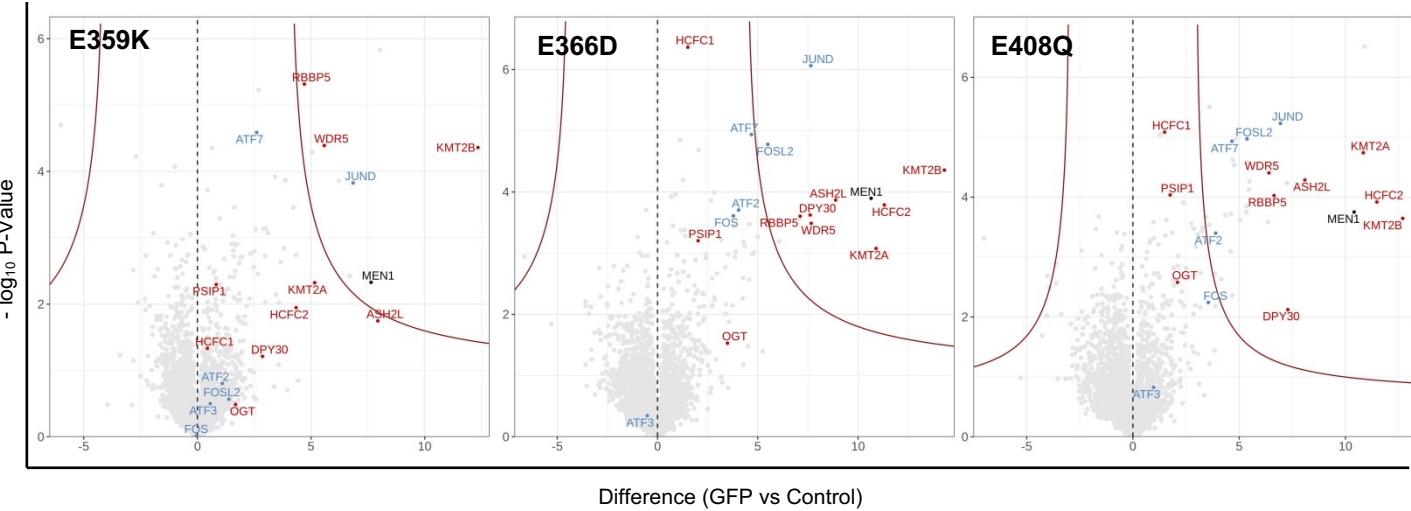

Supplement: Supplementary file 3 — Additional file 3: Figure S3. Volcano plots of interactors of mutant menin-GFP isolated from nuclear extracts. FDR = 0.01, s0 = 2 cutoff was applied to determine significant interactors. The menin-GFP proteins are indicated by a black dot, whereas subunits of MLL1/MLL2 complexes are indicated in red and JunD-containing complexes in blue. Please note that some MLL1/MLL2 complex members score as significant in these Volcano plots, for example with L186P, E255K or L268P. However quantitative analyses based on iBAQ values as provided in Fig. 3B and 3C indicate strongly reduced MLL1/MLL2 interactions of these mutants. [file 13072_2022_461_MOESM3_ESM.pdf]

**A**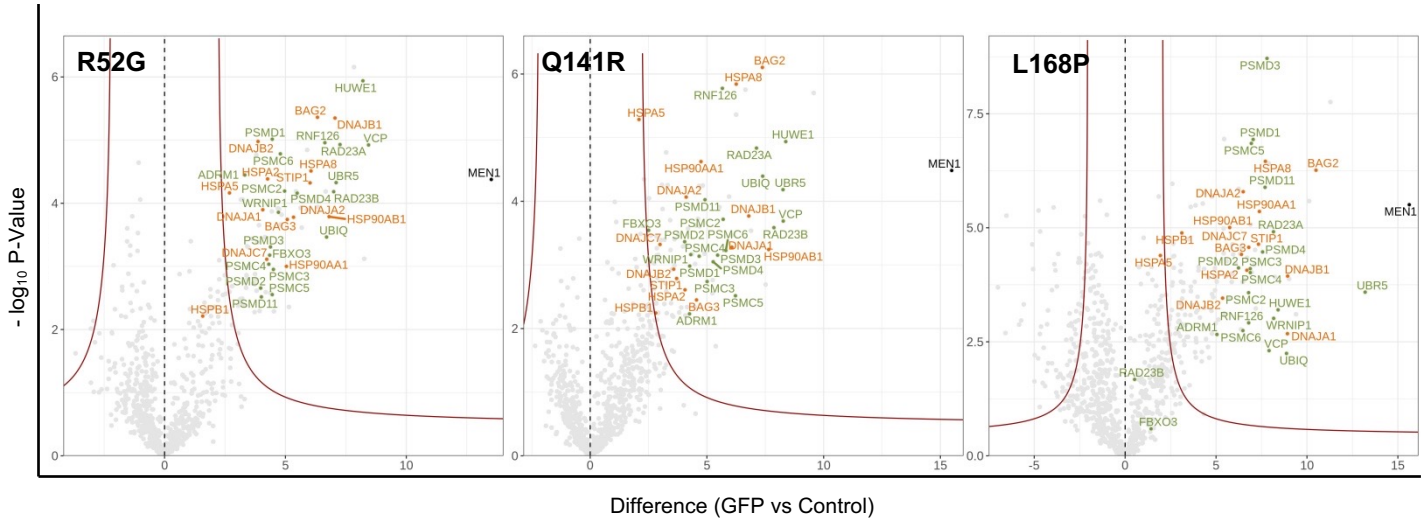**B**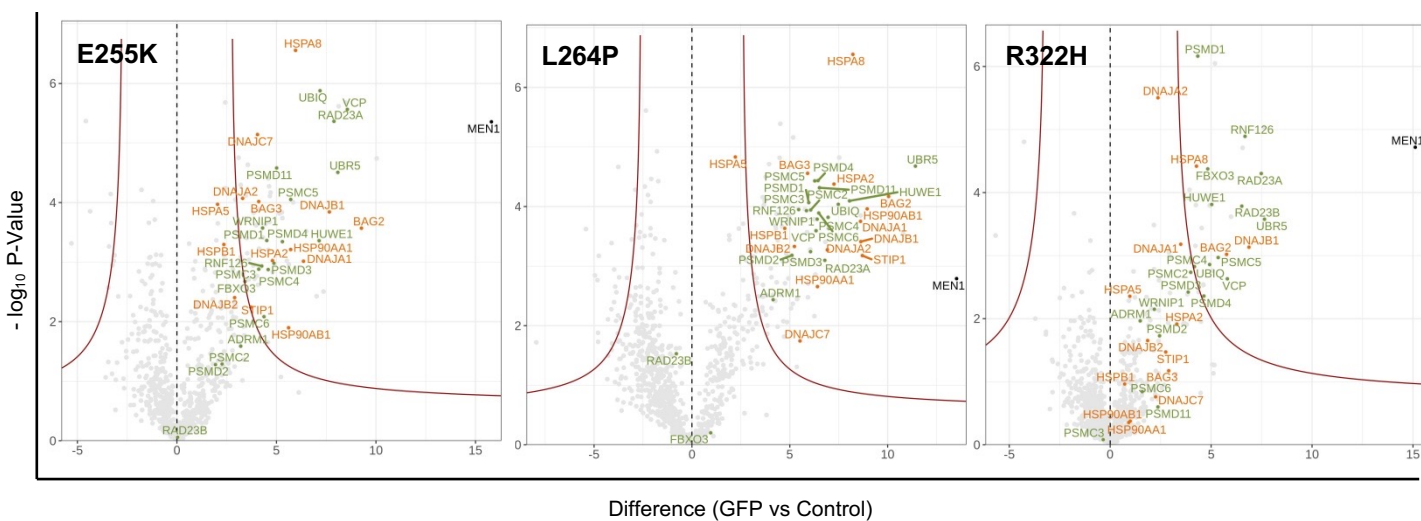**C**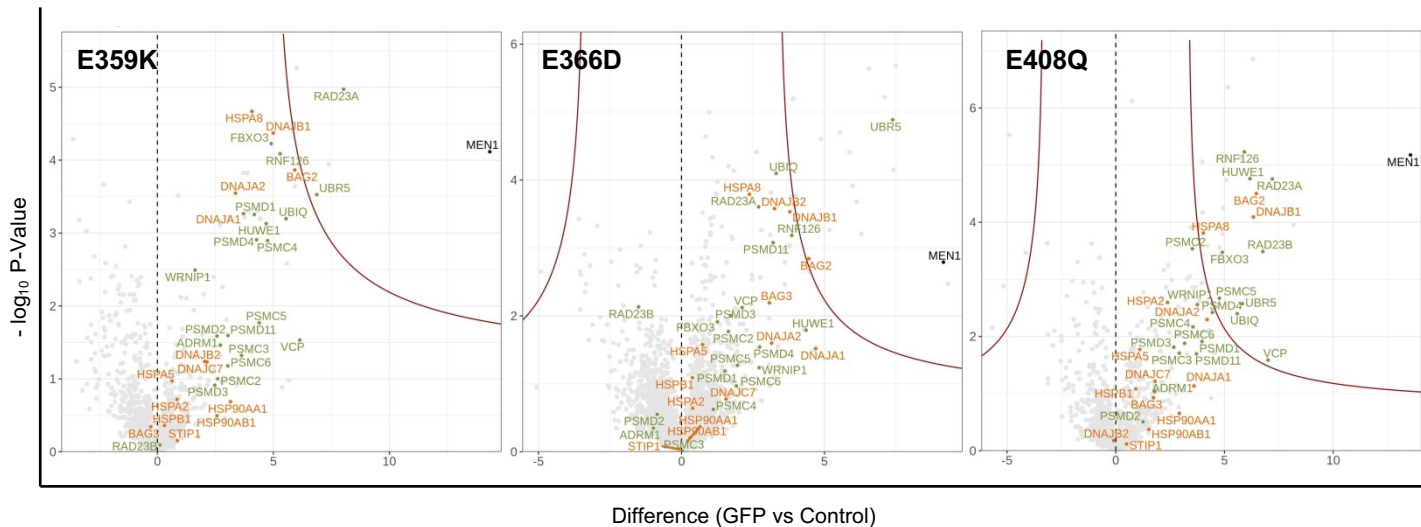

Supplement: Supplementary file 4 — Additional file 4: Figure S4. Volcano plots of interactors of mutant menin-GFP isolated from cytoplasmic extracts. FDR = 0.01, s0 = 2 cutoff was applied to determine significant interactors. The menin-GFP proteins are indicated by a black dot. Proteins involved in ubiquitination are indicated in green and protein chaperones in orange. [file 13072_2022_461_MOESM4_ESM.pdf]
