# Supplementary material for: Multi-omics analyses of MEN1 missense mutations identify disruption of menin–MLL and menin–JunD interactions as critical requirements for molecular pathogenicity
Source: Epigenetics Chromatin. 2022 Aug 9;15:29. doi: 10.1186/s13072-022-00461-8 (PMC9361535; doi:10.1186/s13072-022-00461-8)
Supplement: Supplementary file 6 — Additional file 6: Table S2: Summary of the data sets used in this study. [file 13072_2022_461_MOESM6_ESM.docx]

**Additional file 6: Table S2**: Summary of the data sets

| Study | Encode IDs | accession ID (GEO/SRA) | sample_name | Pair/Single reads (in millions) |
| --- | --- | --- | --- | --- |
| Present study | - | SRR16494839 | MEN1 (wild type) experiment | 7.63992 |
| Present study | - | SRR16494838 | MEN1 (wild type) control | 2.43454 |
| Present study | - | SRR16494831 | MEN1 (E408Q) experiment | 25.5207 |
| Present study | - | SRR16494830 | MEN1 (E408Q) control | 4.849 |
| Present study | - | SRR16494829 | MEN1 (R52G) experiment | 32.38166 |
| Present study | - | SRR16494828 | MEN1 (R52G) control | 5.6406 |
| Present study | - | SRR16494827 | MEN1 (E255K) experiment | 18.78375 |
| Present study | - | SRR16494826 | MEN1 (E255K) control | 4.90653 |
| Present study | - | SRR16494825 | MEN1 (E359K) experiment | 25.03303 |
| Present study | - | SRR16494824 | MEN1 (E359K) control | 5.78129 |
| Present study | - | SRR16494837 | MLL1 experiment | 13.30903 |
| Present study | - | SRR16494836 | MLL1 control | 2.41639 |
| Present study | - | SRR16494835 | JunD experiment | 26.42829 |
| Present study | - | SRR16494834 | JunD control | 6.39683 |
| Present study | - | SRR16494833 | PolII (RPB1) experiment | 5.80083 |
| Present study | - | SRR16494832 | PolII (RPB1) control | 2.11888 |
| - | - | SRR8171284/GSM3462842 | ATACseq | 19.766614 |
| Encode | ENCFF063XTI | GSM733682 | H3K4me3 | 15.810702 |
| Encode | ENCFF617YCQ | GSM798322 | H3K4me1 | 15.505318 |
| Encode | ENCFF113QJM | GSM733684 | H3K27ac | 14.815657 |
